# Assessing Renal Function for Kidney Donation. How Low Is Too Low?

**DOI:** 10.3389/fmed.2021.784435

**Published:** 2022-02-02

**Authors:** Gustavo Laham, Juan Pablo Ponti, Gervasio Soler Pujol

**Affiliations:** Internal Medicine Department, Nephrology Section, Centro de Educación Médica e Investigaciones Clínicas Norberto Quirno (CEMIC), Buenos Aires, Argentina

**Keywords:** end stage kidney disease (ESKD), kidney transplant, glomerular filtration rate, estimated GFR, measure GFR

## Abstract

Kidney transplantation (KT) is the treatment of choice for patients with end-stage kidney disease (ESKD) with decreased morbi-mortality, improved life quality, and reduced cost. However, the shortage of organs from deceased donors led to an increase in KT from living donors. Some stipulate that living donors have a higher risk of ESKD after donation compared with healthy non-donors. The reason for this is not clear. It is possible that ESKD is due to the nephrectomy-related reduction in glomerular filtration rate (GFR), followed by an age-related decline that may be more rapid in related donors. It is essential to assess donors properly to avoid rejecting suitable ones and not accepting those with a higher risk of ESKD. GFR is a central aspect of the evaluation of potential donors since there is an association between low GFR and ESKD. The methods for assessing GFR are in continuous debate, and the kidney function thresholds for accepting a donor may vary according to the guidelines. While direct measurements of GFR (mGFR) provide the most accurate evaluation of kidney function, guidelines do not systematically use this measurement as a reference. Also, some studies have shown that the GFR decreases with age and may vary with gender and race, therefore, the lower limit of GFR in patients eligible to donate may vary based on these demographic factors. Finally, it is known that CrCl overestimates mGFR while eGFR underestimates it, therefore, another way to have a reliable GFR could be the combination of two measurement methods.

## Introduction

The number of patients with end-stage kidney disease (ESKD) requiring dialysis is increasing around the world (1). This has been mainly attributed to the rising prevalence of diabetes mellitus (DM) and hypertension (HTN) in an elder population that reaches this stage (2). Not surprisingly this is associated with increased morbidity, mortality, healthcare costs, and reduced quality of life (3–7). Our country, Argentina, is not an exception; in 2019, there were 30,432 prevalent patients on dialysis with an incidence and a prevalence of 164 and 677 patients per million inhabitants, respectively (8). The annual mortality rate for patients with chronic kidney disease (CKD) stage 5D is 17.3%, being 14.7% for peritoneal dialysis, and 17.7 % for patients on hemodialysis (8).

Today, renal transplantation (RT) is the best treatment option for patients reaching ESKD because it is associated with better patient survival, quality of life, and lower costs compared with patients remaining on dialysis (9). Unfortunately, as time goes by the increasing number of patients reaching ESKD combined with organ shortage makes waiting time for deceased donors RT longer (8). In Argentina, the number of patients on dialysis has increased while the number of RT has remained stable over the years at a rate of 19–21 RT per million inhabitants. The rate increased in 2018 and 2019 to 24.6 and 29.5 per million inhabitants, respectively, at the expenses of deceased donors. However, the percentage of RT with living donors has not increased for the past 10 years, being approximately 20% of all kidney transplants (8).

One strategy to cope with the increasing number of patients on the waiting list is to increase the living kidney donor (LKD) pool with more flexible donor acceptance criteria (10).

One of the major concerns about LKD is whether they have the same mortality and life expectancy compared with a similar age, sex, and comorbidity matched population. In a study by Sergev et al. (11), the risk of death in the first 90 days following live donor nephrectomy was 3.1 per 10,000 donors (95% *CI*, 2.0–4.6). Mortality was higher in men, African Americans, and with a history of HTN. This mortality rate seems low compared with that reported in the literature of laparoscopic cholecystectomy 15/10,000 (12) or nephrectomy of non-donor 260/10,000 surgeries (13). Others studies addressed the long-term risk of death and found that the life expectancy of kidney donors appears to be similar to that of non-donors or perhaps even longer (14, 15). Additionally, Ibrahim et al. (15) compared 3,698 kidney donors' survival who donated kidneys during the period from 1963 through 2007 with the general population from the National Center for Health Statistics. The survival of kidney donors was similar to that of controls who were matched for age, sex, and race or ethnic group. On the other hand, Geir Mjoen et al. (16) analyzed 1,901 subjects who donated kidneys from 1963 to 2007 with a median follow-up of 15.1 years. A control group of 32,621 potentially suitable kidney donors was selected, with a follow-up of 24.9 years. Hazard ratio (*HR*) for all-cause death was significantly increased to 1.30 (95% *CI* 1.11–1.52) for donors compared with controls. One of the reasons that could explain this finding was that the control groups were all from the same county with a higher life expectancy compared with Norway where the donors came from. More recently Shiromani Janki et al. (17) compared 761 LKDs, who visited the outpatient clinic and their propensity score was matched with 1,522 non-donors from population-based cohort studies. The study showed no significant differences between donors and non-donors in overall and cardiovascular mortality.

Another concern in LKD is about the risk of progression to ESKD. Nevertheless, several studies have shown no differences between LKD and matched controls in this respect (16, 18, 19). The study by Ibrahim et al. (15) suggests that there is no excessive risk of ESKD to donors and confirms the view that factors associated with reduced GFR in donors are similar to those that have been observed in the general population. The estimated incidence of ESKD in donors would appear to be 180 per million persons per year, as compared with the overall adjusted incidence rate of 268 per million persons per year in the white population of the United States (15). Between 1996 and 2015, there has been an increase in the number of LKD reaching the United States kidney waiting list with a total of 441 patients (20). In 2014, Muzaale et al. (21) compared 96,217 LKD from the Organ Procurement and Transplantation Network (OPTN) registry (1994–2007) with 20,024 participants of the NHANES III study showing a low incidence of ESKD, 15 years post-donation (estimated risk of 30.8 of 10,000 donors over 15 years). This represented an 8-fold increased incidence of ESKD in kidney donors compared with healthy individuals in the United States who had not donated. This difference was seen in both black and white subjects, with an estimated risk of 74.7 per 10,000 black donors vs. 23.9 per 10,000 black non-donors (*p* < 0.001) and estimated risk of 22.7 per 10,000 white donors vs. 0.0 per white non-donors (*p* < 0.001).

Even though African American (AA) had a higher risk of ESKD in the general population compared with white individuals, the absolute risk of ESKD in black living donors was also low. Finally, the estimated lifetime risk of ESKD was 90 per 10,000 donors, 326 per 10,000 unscreened non-donors (general population), and 14 per 10,000 healthy non-donors (21). The question is whether LKDs develop ESKD as a consequence of new-onset disease that can affect the remaining kidney or is it due to a constant fall in the glomerular filtration rate (GFR) that can occur with aging. Recently a study published by Matas et al. (22) over 40,130 LKDs from 1963 to 2015, 39 developed ESKD, (mean age at ESKD, 62.4 years; mean interval between donation and ESKD, 27.1 ± 9.8 years). Donors who developed ESKD were more likely to be men, as well as smokers, younger at the time of donation, and to have donated to a first-degree relative. Of donors with a known cause of ESKD (*n* = 25), 48% was due to diabetes mellitus (DM) and/or hypertension (HTN), and only two were from a disease that would have affected one kidney (cancer). Therefore, knowing the risk factors for ESKD in LKDs could help improve their selection as well as their medical care after donation. In fact, today LKD tends to be older and have more comorbidities which call for a thorough evaluation before donation (23). O'Keeffe et al. (24) published a systematic review and meta-analysis of 52 studies, including 118,426 LKDs and 117,656 non-donors. They did not find evidence suggesting a higher risk for cardiovascular disease, type 2 diabetes, or adverse psychosocial health outcomes in LKDs than in non-donor populations. On the other hand, kidney donors had higher diastolic blood pressure and a higher risk for preeclampsia in female donors. Although living kidney donation is associated with higher relative risks (RRs) for preeclampsia, the absolute risk for this outcome remains low, compared with non-donor populations. Similar results were obtained by Janki et al. (17), but there was a lower risk of new-onset HTN compared with the meta-analysis (24). Kasiske et al. (25), in a 3-year study, compared 182 donors and 173 non-donors. They found that donors at 3 years follow-up had significantly higher levels of uric acid, phosphorus, and parathyroid hormone (PTH). This could have been related to a decrease in kidney mass in apparently healthy individuals. Whether this has any implications for the bone health of donor is not yet known. In Table 1, we summarized the most relevant studies concerning mortality and progression to ESKD.

**Table 1:** Summary of the most relevant studies related to post-donation medical outcomes.

	**Study design**	**Outcome: Comparison of LKD to Controls**
**Mortality**		
Sergev et al. (11)	80,347 LKD vs. Controls matched from NHANES III by age, gender, race, BMI, smoking history, and SBP, after exclusions for baseline comorbidity	Risk of death in the first 90 days after live donor nephrectomy (3.1/10,000) Long-term LKD mortality not higher vs. matched healthy
Ibrahim et al. (15)	3,698 LKD survival vs. NHANES controls. 1:1 ratio, matched by: age, race and BMI	No differences in mortality between groups
Gier Mjoen et al. (16)	1,901 LKD vs. 32,621 subjects between the ages of 20 and 70, general Norwegian population, with no contraindication to donation of a kidney Matching on age, sex, SBP, BMI and smoking	Risk for LKD: HR for all-cause death: 1.30 (1.11–1.52) and cardiovascular death: 1.40 (1.03–1.91)
Shiromani Janki et al. (17)	761 LKD vs. 1,522 non donors	No differences in overall mortality (OR 0.06, 95% CI 0.05; 0.08) for LKD
**Progression to ESKD**		
Muzaale et al. (21)	96,217 LKD vs. 20,024 patients from the NHANES III study. Excluding those with contraindication to kidney donation: *n* = 9,364 subjects matching on age, sex, race, BMI, smoking and SBP	Incidence of ESKD 30.8/10,000 donors over 15 years vs. 3.9/10,000 in healthy non-donors
Gier Mjoen et al. (16)	1,901 LKD vs. 32,621 subjects between the ages of 20 and 70, general Norwegian population, with no contraindication to donation of a kidney Matching on age, sex, SBP, BMI and smoking	HR for ESKD in LKD: 11.38 (4.37–29.63, *P* < 0.001)
Ibrahim et al. (15)	3,698 LKD survival vs. NHANES controls. 1:1 ratio, matched by: age, race and BMI	No difference in incidence of ESKD: 180 per million persons per year in LKD vs. 268 per million persons per year in controls.
O'Keeffe et al. (24)	Meta-analysis: 52 studies, 118,426 LKD vs. 117,656 controls	LKD: higher diastolic blood pressure, lower estimated glomerular filtration rates. Risk for ESKD (RR, 8.83 [CI, 1.02 to 20.93]), but low absolute risk for ESKD (incidence rate, 0.5 event [CI, 0.1 to 4.9 events] per 1000 person-years)

*LKD, living kidney donor; NHANES III, National Health and Nutrition Examination Study; SBP, Systolic blood pressure; ESKD, end stage kidney disease; BMI, body mass index*.

There is little consensus about what is considered an appropriate GFR threshold for donation and formulation of guidelines is hampered by the heterogeneity of practice in how GFR is measured, uncertainty on the accuracy of GFR estimating equations, and bias and imprecision of standard methods of GFR measurement. The aim of this review is to update the different methods of GFR assessment in live kidney donors, their strengths, and limitations. In addition, we will go through the current guidelines of GFR thresholds available in the literature, and finally, we will analyze the impact of pre-donation GFR on post-donation outcomes.

## Methods

A non-language restricted search was performed until August 30, 2021 in PubMed, SciELO, Trip Database, Google Scholar, and MEDES y MEDLINE, using the following MeSH terms and key words, “living kidney donors,” “chronic kidney disease,” “donor nephrectomy,” “glomerular filtration rate,” “estimate glomerular filtration rate,” “glomerular filtration rate,” “glomerular filtration rate measurements,” “cystatin C,” “CKD-EPI,” “MDRD,” “creatinine clearance,” “inulin,” “iothalamate,” “diethylenetriaminepentaacetic acid,” “iohexol,” and “ethylenediamine tetra-acetic acid.” Bibliographies of relevant articles and reviews were manually screened to identify additional studies.

### GFR in Normal Subjects

A precise assessment of pre-donation GFR and its follow-up is essential to identify donors at risk to initiate prevention strategies.

Glomerular filtration rate has been established for decades by measuring the 24-urine creatinine excretion (26). One of the problems with this method is that in healthy subjects, it overestimates GFR by up to 10% due to tubular excretion of creatinine (27). The others relate to the possible imprecision and bothersome 24 h urine collection. Therefore, other methods to measure GFR (mGFR) have taken place. The exogenous tracers are considered the gold standard (28), such as inulin, technetium-99mTc-diethylenetriaminepentaacetic acid (99mTc-DTPA) (29), 51Cr-ethylenediaminetetraacetic acid (EDTA) (30), 125I-iothalamate (31), and non-reactive Iohexol. Nevertheless, they are expensive, their use is complex, and may have side effects. For this reason, GFR is frequently estimated by equations (eGFR) which incorporate endogenous markers, as well as demographic and anthropometric parameters (27). Among those using creatinine, the chronic kidney disease epidemiology (CKD-EPI) (32) is the most widely used. Other equations include cystatin C (CysC) (33), CysC +Cr (34), beta-trace protein (BTP), and beta2 microglobulin (B2M) (35).

Most of the studies for mGFR and eGFR have been designed and validated in renal patients with chronic kidney disease (CKD) therefore their assessment of GFR in normal individuals, such as LKD, is not precise.

### GFR Measurement

Inulin is a fructose polysaccharide found in the roots of a variety of plants. It has a molecular weight of approximately 5,200 Da. It is neither metabolized nor reabsorbed or secreted by the renal tubules, therefore, it can be quantitatively recovered in the urine after intravenous administration. As described by Homer Smith in 1935 (36), Inulin clearance is constant and independent of its plasmatic concentration. Experimental studies have shown similar concentrations in the Bowman's space and plasma, 99.3% of Inulin injected in the proximal tubule is present in the distal tubule (37). Inulin clearance continues to be the gold standard for the measurement of GFR. Nevertheless, issues concerning its cost, the complexity of the procedure, and scarce availability turns its clinical use generally infeasible. Many procedures to measure mGFR (38) have been developed, all of them are more imprecise than the inulin clearance which is already not 100% precise as it has a coefficient of variation of approximately 7% on repeated measurements in the same subjects (39). One study addressed this issue by comparing the accuracy of mGFR measured by other exogenous tracers to that of inulin. They found that both plasmatic or urinary clearance of 125I-iothalamate, EDTA, or Iohexol, and only the urinary clearance of Tc99-DTPA is sufficiently accurate to measure GFR (40).

Iothalamate is currently used in the United States. It is an ionic contrast tracer with a molecular weight of approximately 637 Da, very little protein-bound (41). It has a good correlation with inulin clearance but as it is secreted and reabsorbed by renal tubules, it has been shown to overestimate GFR (42, 43).

Ethylenediaminetetraacetic acid is available in Europe. Its cost is high as it requires all the precautions for storage, administration, and disposal of radioactive substances. It may have tubular reabsorption as it tends to underestimate GFR compared with inulin (44). Another compound, DTPA is a chelating agent used in the United States and Canada. It produces radiochemical debris while labeling with Tc99. It is protein-bound in about 4–10% increasing its permanence in the circulation which tends to underestimate GFR (45).

Iohexol is a non-ionic, non-radioactive, low-osmolality contrast dye developed in the 1980s (46). It has a molecular weight of 821.1 Da. Its protein-bound is scarce (47). It is cheap, safe, and widely used. Methods taking series of blood samples after tracer injection are popular although not validated for subjects with normal renal function. Only one study measured Iohexol plasma clearance in 20 patients without kidney disease (48). The mGFR obtained after 5 samples (5M) (150/180/200/220/240 min) or 4 samples (4M) (180/200/220/240 min) was compared with that of inulin. The mGFR with the sample taken at 150 min in M5 was the closest to inulin's but still underestimated GFR by 13 ml/min/1.73 m^2^. Authors postulated that if an earlier sample could be more accurate in normal subjects.

### GFR Estimation

Due to the limitations described for mGFR, equations have been developed to estimate GFR (eGFR). As we mentioned they are based on endogenous substances. Creatinine (Cr) is the most widely used. Cr is a 113 Da amino acid generated in the muscle from ingested food. It spreads in total body water, it is filtered by the glomerulus, secreted by the tubules, and excreted in the urine. We will focus on two equations, modification of diet in renal disease (MDRD) and CKD-EPI. None of them were developed in healthy subjects (49). MDRD was developed in 1999 (50) with creatinine standardization by the isotope dilution mass spectrometry in 2006. Compared with Cockroft-Gault, MDRD is more accurate (51). The most important limitation of this formula is that it underestimates GFR with values over 60 ml/min/1.73 m^2^. Different from MDRD, CKD-EPI was validated in a cohort including not only patients with CKD but also individuals with normal renal function which provides a better correlation with normal subjects (52).

The performance of these equations in LKD has been explored. In one study (53), the accuracy of MDRD and CKD-EPI for different clinical situations including LKD (*n* = 583) was evaluated. For the detection of an mGFR < 60 ml/min/1.73 m^2^, CKD-EPI had a sensitivity of 50% and specificity of 98%, while MDRD had 70 and 94%, respectively. For the detection of an mGFR < 80 ml/min/1.73 m^2^, CKD-EPI had a sensitivity of 71% and specificity of 76% while MDRD had 89 and 48%, respectively.

Another study (54) evaluating LKD found that the probability of having an mGFR <80 ml/min/1.73 m^2^ with the eGFR < 80 ml/min/1.73 m^2^ was 14%, in other words, 86% of discharged donors by eGFR would have been donors by mGFR. Therefore, the use of the eGFR with creatinine for LKD selection is flawed by the risk of rejecting candidates with a falsely low eGFR and accepting others with a falsely high eGFR. Another endogenous substance used to estimate GFR is Cystatin C (CysC), a protein that inhibits cysteine and is secreted by the majority of cells. CysC is freely filtered by the glomerulus, almost fully reabsorbed, and metabolized by tubular epithelial cells, it is not secreted and urinary excretion is negligible. Blood levels are less affected than creatinine by body mass, diet, age, or sex (55). Nevertheless, they can be affected by hyperthyroidism, high-dose steroids, and cardiovascular disease (56).

There are several equations available that incorporate CysC either alone or in combination with creatinine to estimate GFR. In patients with CKD, it has been demonstrated that eGFR by CKD-EPI with CysC+Cr has higher precision than CKD-EPI only with Cr (34). One study (57) performed in 147 potential LKD found that the combined equation underestimated mGFR less than the only Cr-based equation (−2,7 vs. −11.6 ml/min/1.73 m^2^). The greater difference was observed with mGFR between 89 and 60 ml/min/1.73 m^2^ where CycC+Cr had −4.3 ml/min/1.73 m^2^ vs. −15 ml/min/1.73 m^2^ for the only Cr based equation.

Nevertheless, there are circumstances where the eGFR + CysC is decreased compared with eGFR + Cr or mGFR. The so-called “shrunken pore syndrome” has been defined when eGFR + CysC is less than 60% of the eGFR + Cr (58). In 2015, it was described in pregnant women in their third trimester (59). It has also been associated with increased mortality after cardiac surgery (60) and decreased left ventricular systolic function (61). Its relevance to LKD has not been yet evaluated.

A Spanish group (62) compared mGFR by iohexol with that of 51 different equations based on Cr, CysC or both, in the selection of 103 LKD. The threshold for donation was established at mGFR > 80 ml/min for those >35 years old or 90 ml/min for those <35 years old. In total, 93 subjects (90.3%) had mGFR over the threshold and 10 (9.7%) below the threshold. Many of those not selected by mGFR were over the threshold by eGFR and would have been selected. All of those excluded were women. In subjects selected by mGFR, 32 were below the threshold by eGFR that would have left them out for donation.

### Guidelines for GFR Evaluation Before LKD

Several guidelines are addressing this matter. They use different methods to evaluate GFR and different GFR thresholds. We will summarize the ones we considered the most relevant (Table 2).

**Table 2:** Guidelines for glomerular filtration rate (GFR) evaluation before living kidney donation.

	**KDIGO**	**BTS**		**CTS**	**OPTN**
Initial evaluation	CKD-EPI+Cr	CKD-EPI+Cr		eGFR Cr (CKD-EPI/Cockcroft-Gault)	mGFR or CrCl
Confirmation	Depending on availability: -mGFR (exogenous or endogenous markers) -eGFR cycs+cr -repeat eGFR Cr	mGFR (inulin, 51Cr-EDTA, 125I-iothalamate or iohexol)		Two separate creatinine clearance or one mGFR (DTPA, EDTA, iohexol or iothalamate)	—
Threshold GFR (ml/min/1,73 m2)	Accept: >90 Exclude: < 60 Individualize decisions: 89–60	Accept: Male: 20–29yrs: >90 30–55yrs: >80 60yrs: >76 65yrs: >71 70yrs: >67 75yrs: >63 80yrs: >58	Female: 20–29yrs: >90 30–50yrs: >80 55yrs: >75 60yrs: >70 65yrs: >64 70yrs: >59 75yrs: >54 80yrs: >49	Accept: 18–30yrs: ≥90 31–40yrs: ≥85 41–65yrs: ≥80 >65yrs: ≥75	Accept: CrCl > 80 or predicted GFR at 80yrs: > 40
Others	Web-based calculator to estimate the probability of having a mGFR below 60, 70, 80 and 90 mL/min/1.73 m2	Size difference > 10%: mGFR 51Cr-EDTA + 99mTc-DTPA. Donate the least functioning		Size difference > 1 cm: mGFR 99mTc-DTPA. If differential GFR is more than 5%: Donate the least functioning	—

### Kidney Disease: Improving Global Outcomes-KDIGO 2017

The initial recommended test is CKD-EPI + Cr, and then confirmatory testing, as needed. Depending on availability, mGFR, with exogenous or endogenous markers, eGFR combining CysC + Cr or repeating an eGFR Cr can be used (63).

Another tool suggested by the guideline is a web-based calculator to estimate the probability of having an mGFR below 60, 70, 80, and 90 ml/min/1.73 m^2^ (http://ckdepi.org/equations/donor-candidate-GFR-calculator/). It is divided into two steps. First, it calculates the pre-test probability of having an mGFR below 60, 70, 80, and 90 ml/min/1.73 m^2^ based on gender, age, and ethnicity. It then performs a post-test taking into account creatinine measurements with or without cystatin, using data obtained from the CKD-EPI cohort and eGFR/mGFR concordance. For example, a 25-year-old white male with a plasma creatinine of 1 mg/dl (eGFR CKD-EPI 104 ml/min/1.73 m^2^) has a post-test probability of having an mGFR of less than 90 ml/min/1.73 m^2^ of 3%.

To accept a candidate routinely, they suggest an eGFR > 90 ml/min/1.73 m^2^ and < 60 ml/min/1.73 m^2^ to exclude the participant. For values in the middle decisions should be individualized and other risk factors should be considered. This recommendation was based on a meta-analysis with almost 5 million healthy subjects where they found that for an eGFR > 90 ml/min/1.73 m^2^, the life-long risk of developing CKD was approximately 1% of any age and race. For subjects aged 60 years or older with an eGFR between 60 and 89 ml/min/1.73 m^2^, the risk is less than 1% (64).

### British Transplantation Society-BTS 2018

Similar to KDIGO, they recommend CKD-EPI + Cr as the initial test. This will identify and rule out potential donors with CKD (eGFR < 45 ml/min/1.73 m^2^). For the rest, GFR should be confirmed by mGFR with inulin clearance, 51Cr-EDTA, 125I-iothalamate, or iohexol (65).

Whenever there is a disparity between the two kidneys greater than 10%, they recommend performing a differential mGFR for each kidney with a combination of 51Cr-EDTA and 99mTc-DMSA. If eligible, the less functioning kidney should be donated.

Glomerular filtration rate thresholds for donation should be adapted for age and sex. A study performed in more than 1,800 potential LKDs found that until age of 40 years, GFR remained stable, thereafter it decreased 6.6 ml/min/1.73 m^2^ per decade in men and 7.7 ml/min/1.73 m^2^ in women (66). In patients older than 35 years, a GFR >80 ml/min/1.73 m^2^ seems safe for donation. In those younger than 30 years, a more conservative approach is warranted as there is evidence of a greater risk of CKD in this population with GFR < 90 ml/min/1.73 m^2^ (64).

### Canadian Transplant Society-CTS 2015

The recommendations include 2 creatinine determinations along with CKD-EPI or Cockcroft-Gault eGFR. In addition, 2 separate creatinine clearance measurements or one mGFR by DTPA, EDTA, iohexol, or iothalamate (67).

Additionally, it is suggested that asymmetric kidneys (>1 cm) should be evaluated by 99mTc-DTPA mGFR. If differential GFR is more than 5%, the donor should be left with the best functioning kidney.

Glomerular filtration rate thresholds are dependent on age. Potential donors between 18 and 30 years old should have a GFR ≥ 90 ml/min/1.73 m^2^ to be accepted, a GFR ≥ 85 ml/min/1.73 m^2^ for donors between 31 and 40 years old, a GFR ≥ 80 ml/min/1.73 m^2^ for those 41 to 65 years old, and a GFR ≥ 75 ml/min/1.73 m^2^ for those >65 years old.

### Organ Procurement and Transplantation Network-OPTN

In their guidelines, they propose mGFR using exogenous filtration markers or creatinine clearance.

They suggest that those with a creatinine clearance < 80 ml/min/1.73 m^2^ (68), or a predicted GFR at age 80 years is < 40 ml/min/1.73 m^2^ (69), should be excluded for dotation (70).

### Which Are the Recommended Methods for the Assessment of Renal Function for the Selection of Kidney Donors

In nephrology, there are few situations where an accurate assessment of GFR is essential, kidney donation is one of them (71), but guidelines do not specify which method should be used (72). In addition, the GFR threshold for kidney donation matters (73). A study carried out in 2007 found that 90% of living kidney transplant programs in the United States used 24-h CrCl (74). This is not an exception in our country. A survey conducted in 28 transplant centers in Argentina showed that 78.5% used CrCl for the assessment of donor's kidney function, while others used eGFR or iothalamate Cl. To accept a kidney donor, a CrCl > 80 ml/min/1.73 m^2^ was required by 71.4% of the physicians while 21.4% required CrCl > 90 ml/min/1.73 m^2^ (Maldonado et al., SLAH congress). It is well known that CrCl overestimates GFR and can lead to the approval of donors with a lower GFR than the optimal adjusted for age and sex.

Many transplant programs around the world use other methods to measure kidney function, such as eGFR or mGFR. The availability of mGFR varies, especially in developing countries where health resources are limited and where living kidney donation can be the only source of kidney transplants (75). On the other hand, the use of eGFR in the evaluation and selection of LKDs is controversial, in part due to concerns about its accuracy and correlation with mGFR to accept or reject donors (76). An alternative approach is to adapt eGFR thresholds to the best locally available technique. Two recently published studies offer a web-based application using eGFR to compute the probability that the measured GFR of a donor candidate is higher than the threshold previously defined at 80 ml/min/1.73 m^2^ (77, 78). These studies indicate that it is possible to define different eGFR thresholds for mGFR limits coping with a certain degree of uncertainty as to whether the mGFR threshold is reached. In agreement with these findings, the KDIGO Clinical Practice Guideline for LKD evaluation recommends using eGFR as a test to identify candidates who may not need a subsequent GFR assessment (63).

Previous research comparing eGFR and mGFR in living donors showed that suitable donors with a falsely low eGFR could have been accepted as well as inappropriate donors with a falsely high eGFR should have been rejected (54, 76).

Gaillard et al. (72) evaluated 2,733 donors from 11 French transplant centers. They examined whether relying on an eGFR rather than mGFR measurement alters the choice of potential living donors by comparing the effect of 4 equations (MDRD, CKD-EPI, Lund Malmö equation, and full age spectrum [FAS] equation) with mGFR as the reference method. Additionally, they studied the impact of using absolute or age-adapted GFR thresholds. They found that the CKD-EPI and FAS equations had the best performances and led to the lowest percentage of inappropriately evaluated candidates. Misclassification was more frequent when GFR adequacy was defined as an absolute threshold of 90 ml/ min/1.73 m^2^ as compared with an age-adapted definition (26 and 5%, respectively). Accepting an absolute eGFR threshold of 90 ml/min/1.73 m^2^, 1,804 potential donors were identified, compared with 2,648 when mGFR was interpreted using age-adjusted thresholds. They strongly suggest that mGFR should be the gold standard for donor evaluation, but in cases where eGFR is the only source of measurement, age-adapted GFR values estimated with either the CKD-EPI or FAS equations should be used.

Recently, Garg et al. (71) compared 1,412 donors in the performance of eGFR with CKD-EPI equation, 24-h CrCl, and the average of these 2 measurements (Avg [CrCl and eGFR] with mGFR by 125I-iothalamate as the gold standard). They found that 24-h CrCl overestimated iothalamate GFR (iGFR) in the entire cohort with an average bias of 2.2 ml/min/1.73 m^2^. However, in men, CrCl overestimated mGFR by 6.7 ml/min/1.73 m^2^, and in women, CrCl slightly underestimated GFR by 1 ml/min/1.73 m^2^. eGFR underestimated iGFR with a median bias of −5.4 ml/min/1.73 m^2^. Results were similar regardless of age and gender, however, in black potential donors, eGFR overestimated iGFR by 3.2 ml/min/1.73 m^2^. Among the three testing GFR methods, median bias was significantly lower using Avg (CrCl and eGFR) at −1.0 ml/min/1.73 m^2^. They concluded that these 2 GFR testing methods could provide a reference in clinical practice, as all transplant programs have access to them.

### Renal Function After Donation

What happens with GFR after donation? With approximately 50% of the renal mass, the remaining kidney undergoes compensatory hypertrophy, and approximately 6 months after donation, GFR returns to 70% of the pre-donation values (79).

We have already addressed the literature regarding the risk of ESKD after donation but little attention has been paid to intermediate-risk factors that lead to it, such as DM, HTN, or impaired renal function. Ibrahim et al. (80) studied 3,956 LKDs followed for more than 40 years after donation. development of ESKD, reduced GFR, reduced eGFR (< to 30, 45, and 60 ml/min/1.73 m^2^) and proteinuria were analyzed. After a mean follow-up of 16.6 ± 11.9 years, 6.1% developed proteinuria after donation. This was associated with a higher body mass index (BMI) (*HR*, 1.10; 95% *CI*, 1.06–1.13; *p* < 0.001) and male gender (*HR*, 1.56; 95% *CI*, 1.18–2.05; *p* < 0.001). Regarding renal function, 35.6% had an eGFR < 60 ml/min/1.73 m^2^ at a median age of 56.6 years and a median time of 9.2 years from the donation. Risk factors associated with an eGFR < 60 were older age at donation, higher BMI, a higher baseline systolic blood pressure, and type 2 DM in the recipient. Additionally, 10.9% had an eGFR < 45 ml/min/1.73 m^2^, older age at donation, higher BMI, and higher baseline systolic blood pressure were their risk factors. Finally, 2.6% had an eGFR < 30 ml/min/1.73 m^2^ at a median age of 68.4 years and after a median time of 23.9 years after donation. This was associated with older age at donation and higher BMI. Interestingly, this study showed that a higher pre-donation eGFR and younger donor age was associated with better post-donation eGFR even after 40 years after donation. In multivariate time-dependent analysis, post-donation diabetes, new-onset hypertension, proteinuria, and eGFR < to 60 or 45 ml/min/1.73 m^2^, were potent predictors of eGFR < 30 ml/min or ESKD.

In previous studies (16, 21), it has been shown that most of the donors developing ESKD donated their kidney to a relative. It is well known that relatives of a patients with ESKD have a higher risk of ESKD (81). Matas et al. (22) tried to determine whether donors having a relative with ESKD had a faster decline in eGFR compared with those without ESKD. They compared long-term post-donation eGFR trajectory for donors with (*n* = 1,245) vs. without (*n* = 757) a first-degree relative with ESKD. After adjusting for other patient factors, donors with a first-degree relative with ESKD had either a smaller increase or a larger decrease in eGFR—on average 0.20 ml/min/1.73 m^2^/year (0.07–0.33) more than donors without a first-degree relative with ESKD, in a wide age range. The authors suggest that, for donors with and without a first-degree relative with ESRD, there is a steady increase in GFR for the first few year's post-donation. Although there was a small difference between the slope of GFR in donors with (vs. without) a first-degree relative with ESRD, neither group experienced a decline in GFR that would explain any increased incidence of ESRD.

A European study from the Netherlands, that we have already mentioned above (17), showed that in kidney donors after mean follow of more than 8 years, there were increase in serum creatinine of 26 μmol/l (95% *CI* 24–28), a decrease in eGFR of 27 ml/min/1.73 m^2^ (95% *CI* −29 to −26), and an eGFR decline of 32% (95% *CI* 30–33) compared with non-donors. There were no differences between groups concerning ESKD. Recently, Agustine et al. (79) evaluated in a cohort of 34,505 LKDs from the Scientific Registry of Transplant Recipients (SRTR) database, factors associated with post-donation renal function and proteinuria. They found an overall median decline of eGFR of 31.1%. Nevertheless, 74% of donors older than 60 years had an eGFR < 60 ml/min 2 years after donation. At that time, they found that older donor age, male gender, black race, HTN, and a BMI > 25 kg/m^2^ were associated with greater decline of eGFR. Although they found that incident proteinuria was independently associated with black race, male gender, and higher BMI, they could not find a correlation between proteinuria and eGFR decline 2 years after donation.

Finally, Gaillard et al. (82) in 2021 studied 1,825 French LKDs. After a mean follow-up of almost 6 years, they found that in donors younger than 45 years post-donation eGFR, absolute- and relative-eGFR variation were not different among the three groups, normal for age (Sage), higher than 90 (S90), or 80(S80) ml/min/1.73 m^2^. However, for older donors, eGFR after donation was higher in S90 than in S80 or Sage. They concluded that donors with a normal eGFR for age (Sage) are older than donors with an eGFR ≥ 90 ml/min/1.73 m^2^ (S90). Differences in GFR after donation are partly attributable to this age difference. They suggest a depth screening for all donor candidates with a normal eGFR for age.

## Conclusions

Currently, the best way to assess the kidney function of donor remains a matter of discussion. The available guidelines for kidney donation are purely based on opinion, and they are not evidence-based. Several methods are clinically available and recommended for living donor evaluation, but each has its drawbacks. Moreover, most of them have been only validated for CKD population. mGFR remains the gold standard, but it is not available worldwide. Therefore, GFR at donation should be considered in the context of normal GFR level based on gender and age. In our experience and that of others, a combination of 2 methods, such as a CrCl and eGFR, may be more clinically useful. Transplant programs are responsible for developing a threshold to accept or deny a living donor based on lifetime risk of kidney failure and apply them uniformly.

Finally, renal function recovery is variable and data suggests that GFR recovery, and post-donation GFR, is associated with donor age, BMI, gender, HTN, and family history.

## Author Contributions

All authors listed have made a substantial, direct, and intellectual contribution to the work and approved it for publication.

## Conflict of Interest

The authors declare that the research was conducted in the absence of any commercial or financial relationships that could be construed as a potential conflict of interest.

## Publisher's Note

All claims expressed in this article are solely those of the authors and do not necessarily represent those of their affiliated organizations, or those of the publisher, the editors and the reviewers. Any product that may be evaluated in this article, or claim that may be made by its manufacturer, is not guaranteed or endorsed by the publisher.

## References

[1] Zhang QL Rothenbacher D. Prevalence of chronic kidney disease in population-based studies: Systematic review. BMC Public Health. (2008) 8:1–3. 10.1186/1471-2458-8-117 18405348 PMC2377260

[2] Liyanage T Ninomiya T Jha V Neal B Patrice HM Okpechi I. Worldwide access to treatment for end-stage kidney disease: a systematic review. Lancet. (2015) 385:1975–82. 10.1016/S0140-6736(14)61601-9 25777665

[3] Manns BJ Mendelssohn DC Taub KJ. The economics of end-stage renal disease care in Canada: incentives and impact on delivery of care. Int J Health Care Finance Econ. (2007) 7:149–69. 10.1007/s10754-007-9022-y 17641968

[4] Klarenbach S Gill JS Knoll G Caulfield T Boudville N Prasad GV. Donor Nephrectomy Outcomes Research (DONOR) Network. Economic consequences incurred by living kidney donors: a Canadian multi-center prospective study. Am J Transplant. (2014) 14:916–22. 10.1111/ajt.12662 24597854 PMC4285205

[5] Canadian Institute for Health Information. Report: CORR 2016. (2016). Available online at: https://www.cihi.ca/en/organ-replacements/corrannual-report. (accessed June 1, 2018).

[6] Laupacis A Keown P Pus N Krueger H Ferguson B Wong C. A study of the quality of life and cost-utility of renal transplantation. Kidney Int. (1996) 50:235–42. 10.1038/ki.1996.307 8807593

[7] Klarenbach SW Tonelli M Chui B Manns BJ. Economic evaluation of dialysis therapies. Nat Rev Nephrol. (2014) 10:644–52. 10.1038/nrneph.2014.145 25157840

[8] Marinovich S Bisigniano L Hansen Krogh D Celia E Tagliafichi V Rosa Diez G. Registro Argentino de Diálisis Crónica SAN-INCUCAI 2019. Sociedad Argentina de Nefrología e Instituto Nacional Central Único Coordinador de Ablación e Implante Buenos Aires, Argentina. (2020).

[9] Wolfe RA Ashby VB Milford EL Ojo AO Ettenger RE Agodoa LY. Comparison of mortality in all patients on dialysis, patients on dialysis awaiting transplantation, and recipients of a first cadaveric transplant. N Engl J Med. (1999) 341:1725–30 10.1056/NEJM199912023412303 10580071

[10] Reese PP Feldman HI McBride MA Anderson K Asch DA Bloom RD. Substantial variation in the acceptance of medically complex live kidney donors across US renal transplant centers. Am J Transplant. (2008) 8:2062–70. 10.1111/j.1600-6143.2008.02361.x 18727695 PMC2590588

[11] Sergev DL Muzaale AD Caffo BS Mehta SH Singer AL Taranto SE. Perioperative mortality and long-term survival following live kidney donation. JAMA. (2010) 303:959–66. 10.1001/jama.2010.237 20215610

[12] Steiner CA Bass EB Talamini MA Pitt HA Steinberg EP. Surgical rates and operative mortality for open and laparoscopic cholecystectomy in Maryland. N Engl J Med. (1994) 330:403–8. 10.1056/NEJM199402103300607 8284007

[13] Birkmeyer JD Siewers AE Finlayson EV Stukel TA Lucas FL Batista I. Hospital volume and surgical mortality in the United States. N Engl J Med. (2002) 346:1128–37. 10.1056/NEJMsa012337 11948273

[14] Fehrman-Ekholm I Elinder CG Stenbeck M Tydén G Groth CG. Kidney donors live longer. Transplantation. (1997) 64:976–8. 10.1097/00007890-199710150-00007 9381544

[15] Ibrahim HN Foley R Tan L Rogers T Bailey RF Guo H. Long-term consequences of kidney donation. N Engl J Med. (2009) 360:459–69. 10.1056/NEJMoa0804883 19179315 PMC3559132

[16] Mjøen G Hallan S Hartmann A Foss A Midtvedt K Øyen O. Long-term risks for kidney donors. Kidney Int. (2014) 86:162–7. 10.1038/ki.2013.460 24284516

[17] Janki S Dehghan A van de Wetering J Steyerberg EW Klop KWJ Kimenai HJAN. Long-term prognosis after kidney donation: a propensity score matched comparison of living donors and non-donors from two population cohorts. Eur J Epidemiol. (2020) 35:699–707. 10.1007/s10654-020-00647-y 32440788 PMC7387377

[18] Okamoto M Akioka K Nobori S Ushigome H Kozaki K Kaihara S. Short-and long-term donor outcomes after kidney donation: analysis of 601 cases over a 35-year period at Japanese single center. Transplantation. (2009) 87:419–23. 10.1097/TP.0b013e318192dc95 19202449

[19] Fournier C Pallet N Cherqaoui Z Pucheu S Kreis H Méjean A. Very long-term follow-up of living kidney donors. Transpl Int. (2012) 25:385–90. 10.1111/j.1432-2277.2012.01439.x 22356210

[20] Matas AJ Hays RE Ibrahim HN. A case-based analysis of whether living related donors listed for transplant share ESRD causes with their recipients. Clin J Am Soc Nephrol. (2017) 12:663–8. 10.2215/CJN.11421116 28249957 PMC5383394

[21] Muzaale AD Massie AB Wang MC Montgomery RA McBride MA Wainright JL. Risk of end-stage renal disease following live kidney donation. JAMA. (2014) 311:579–86. 10.1001/jama.2013.285141 24519297 PMC4411956

[22] Matas AJ Vock DM Ibrahim HN. GFR. ≤ 25 years postdonation in living kidney donors with (vs without) a first-degree relative with ESRD. Am J Transplant. (2018) 18:625–31. 10.1111/ajt.14525 28980397 PMC5820146

[23] LaPointe Rudow D Warburton KM. Selection and postoperative care of the living donor. Med Clin North Am. (2016) 100:599–611. 10.1016/j.mcna.2016.01.009 27095648

[24] O'Keeffe LM Ramond A Oliver-Williams C Willeit P Paige E Trotter P. Mid- and long-term health risks in living kidney donors: a systematic review and meta-analysis. Ann Intern Med. (2018) 168:276–84. 10.7326/M17-1235 29379948

[25] Kasiske BL Anderson-Haag T Israni AK Kalil RS Kimmel PL Kraus ES. prospective controlled study of living kidney donors: three-year follow-up. Am J Kidney Dis. (2015) 66:114–24. 10.1053/j.ajkd.2015.01.019 25795073 PMC4485526

[26] Levey AS. Measurement of renal function in chronic renal disease. Kidney Int. (1990) 38:167–84. 10.1038/ki.1990.182 2200925

[27] Pérez Loredo J Lavorato CA Negri AL. Tasa de filtración glomerular medida y estimada. Numerosos métodos de medición (Parte I). Rev Nefrol Dial Traspl. (2017) 35:153–64. Available online at: https://www.revistarenal.org.ar/index.php/rndt/article/view/34

[28] Eurotransplant Statistical Report 2017. Available online at https://www.eurotransplant.org/cms/index.php?page=sr2017. (accessed February 9, 2019).

[29] Ribstein J du Cailar G Mimran A. Combined renal effects of overweight and hypertension. Hypertension. (1995) 26:610–5. 10.1161/01.HYP.26.4.610 7558220

[30] Nielsen S Rehling M Schmitz A Mogensen CE. Validity of rapid estimation of glomerular filtration rate in type 2 diabetic patients with normal renal function. Nephrol Dial Transplant. (1999) 14:615–9. 10.1093/ndt/14.3.615 10193808

[31] McLeay SC Morrish GA Kirkpatrick CM Green B. Encouraging the move towards predictive population models for the obese using propofol as a motivating example. Pharm Res. (2009) 26:1626–34. 10.1007/s11095-009-9873-7 19337821

[32] Documento de consenso sobre la Enfermedad Renal Crónica. Available online at: http://www.senefro.org/modules/news/images/v._5.doc_consenso_final___131212_copy1.pdf. (accessed Noviembre 27, 2012)

[33] Thomassen SA Johannesen IL Erlandsen EJ Abrahamsen J Randers E. Serum cystatin C as a marker of the renal function in patients with spinal cord injury. Spinal Cord. (2002) 40:524–8. 10.1038/sj.sc.3101320 12235535

[34] Inker LA Schmid CH Tighiouart H Eckfeldt JH Feldman HI Greene T. CKD-EPI Investigators. Estimating glomerular filtration rate from serum creatinine and cystatin C. N Engl J Med. (2012) 367:20–9. 10.1056/NEJMoa1114248 22762315 PMC4398023

[35] Filler G Priem F Lepage N Sinha P Vollmer I Clark H. Beta-trace protein, cystatin C, beta(2)-microglobulin, and creatinine compared for detecting impaired glomerular filtration rates in children. Clin Chem. (2002) 48:729–36. 10.1093/clinchem/48.5.729 11978599

[36] Shannon JA Smith HW. The excretion of inulin, xylose and urea by normal and phlorizinized man. J Clin Invest. (1935) 14:393–401. 10.1172/JCI100690 16694313 PMC424694

[37] Marsh D. Frasier C: Reliability of inulin for determining volume flow in rat renal cortical tubules. Am J Physiol. (1965) 209:283–6. 10.1152/ajplegacy.1965.209.2.283 14321123

[38] Levin A Stevens PE Bilous RW Coresh J De Francisco AL De Jong PE. Kidney Disease: Improving Global Outcomes (KDIGO). KDIGO 2012 clinical practice guideline for the evaluation and management of chronic kidney disease. Kidney Int. Suppl. (2013) 3:1–150.

[39] Davies DF Shock NW. The variability of measurement of insulin and diodrast tests of kidney function. J Clin Invest. (1950) 29:491–5. 10.1172/JCI102285 15415453 PMC436085

[40] Soveri I Berg UB Björk J Elinder CG Grubb A Mejare I. Review Group. Measuring GFR: a systematic review. Am J Kidney Dis. (2014) 64:411–24. 10.1053/j.ajkd.2014.04.010 24840668

[41] Anderson CF Sawyer TK Cutler RE. Iothalamate sodium I 125 vs cyanocobalamin Co 57 as a measure of glomerular filtration rate in man. JAMA. (1968) 204:653–6. 10.1001/jama.1968.03140210007002 5694506

[42] Odlind B Hällgren R Sohtell M Lindström B. Is 125I iothalamate an ideal marker for glomerular filtration? Kidney Int. (1985) 27:9–16. 10.1038/ki.1985.3 3920429

[43] Botev R Mallié JP Wetzels JF Couchoud C Schück O. The clinician and estimation of glomerular filtration rate by creatinine-based formulas: current limitations and quo vadis. Clin J Am Soc Nephrol. (2011) 6:937–50. 10.2215/CJN.09241010 21454722

[44] Rehling M Møller ML Thamdrup B Lund JO Trap-Jensen J. Simultaneous measurement of renal clearance and plasma clearance of 99mTc-labelled diethylenetriaminepenta-acetate, 51Cr-labelled ethylenediaminetetra-acetate and inulin in man. Clin Sci (Lond). (1984) 66:613–9. 10.1042/cs0660613 6423339

[45] De Santo NG Anastasio P Cirillo M Santoro D Spitali L Mansi L. Measurement of glomerular filtration rate by the 99mTc-DTPA renogram is less precise than measured and predicted creatinine clearance. Nephron. (1999) 81:136–40. 10.1159/000045268 9933747

[46] Olsson B Aulie A Sveen K Andrew E. Human pharmacokinetics of iohexol: a new nonionic contrast medium. Invest Radiol. (1983) 18:177–82. 10.1097/00004424-198303000-00015 6408018

[47] Skinnemoen K. Physicochemical properties and degree of protein binding of iopentol. Acta Radiol Suppl. (1987) 370:33–6. 2980308

[48] Sterner G Frennby B Mansson S Nyman U Van Westen D Almén T. Determining 'true' glomerular filtration rate in healthy adults using infusion of inulin and comparing it with values obtained using other clearance techniques or prediction equations. Scand J Urol Nephrol. (2008) 42:278–85. 10.1080/00365590701701806 17943640

[49] Levey AS Greene T Schluchter MD Cleary PA Teschan PE Lorenz RA. Glomerular filtration rate measurements in clinical trials. Modification of diet in renal disease study group and the diabetes control and complications trial research group. J Am Soc Nephrol. (1993) 4:1159–71. 10.1681/ASN.V451159 8305642 PMC2866096

[50] Levey AS Bosch JP Lewis JB Greene T Rogers N Roth D. more accurate method to estimate glomerular filtration rate from serum creatinine: a new prediction equation. Modification of diet in renal disease study group. Ann Intern Med. (1999) 130:461–70. 10.7326/0003-4819-130-6-199903160-00002 10075613

[51] Stevens LA Manzi J Levey AS Chen J Deysher AE Greene T. Impact of creatinine calibration on performance of GFR estimating equations in a pooled individual patient database. Am J Kidney Dis. (2007) 50:21–35. 10.1053/j.ajkd.2007.04.004 17591522

[52] Bhuvanakrishna T Blake GM Hilton R Burnapp L Sibley-Allen C Goldsmith D. Comparison of estimated GFR and measured GFR in prospective living kidney donors. Int Urol Nephrol. (2015) 47:201–8. 10.1007/s11255-014-0859-y 25374260

[53] Murata K Baumann NA Saenger AK Larson TS Rule AD Lieske JC. Relative performance of the MDRD and CKD-EPI equations for estimating glomerular filtration rate among patients with varied clinical presentations. Clin J Am Soc Nephrol. (2011) 6:1963–72. 10.2215/CJN.02300311 21737852 PMC3156428

[54] Tent H Rook M Stevens LA van Son WJ van Pelt LJ Hofker HS. Renal function equations before and after living kidney donation: a within-individual comparison of performance at different levels of renal function. Clin J Am Soc Nephrol. (2010) 5:1960–8. 10.2215/CJN.08761209 20616162 PMC3001772

[55] Knight EL Verhave JC Spiegelman D Hillege HL de Zeeuw D Curhan GC. Factors influencing serum cystatin C levels other than renal function and the impact on renal function measurement. Kidney Int. (2004) 65:1416–21. 10.1111/j.1523-1755.2004.00517.x 15086483

[56] Herget-Rosenthal S Bökenkamp A Hofmann W. How to estimate GFR-serum creatinine, serum cystatin C or equations? Clin Biochem. (2007) 40:153–61. 10.1016/j.clinbiochem.2006.10.014 17234172

[57] Meeusen JW Rule AD Voskoboev N Baumann NA Lieske JC. Performance of cystatin C- and creatinine-based estimated glomerular filtration rate equations depends on patient characteristics. Clin Chem. (2015) 61:1265–72. 10.1373/clinchem.2015.243030 26240296 PMC4726452

[58] Almén MS Björk J Nyman U Lindström V Jonsson M Abrahamson M. Shrunken pore syndrome is associated with increased levels of atherosclerosis-promoting proteins. Kidney Int Rep. (2018) 4:67–79. 10.1016/j.ekir.2018.09.002 30596170 PMC6308389

[59] Grubb A Lindström V Jonsson M Bäck SE Åhlund T Rippe B. Reduction in glomerular pore size is not restricted to pregnant women. Evidence for a new syndrome: ‘Shrunken pore syndrome’. Scand J Clin Lab Invest. (2015) 75:333–40. 10.3109/00365513.2015.1025427 25919022 PMC4487590

[60] Dardashti A Nozohoor S Grubb A Bjursten H. Shrunken Pore Syndrome is associated with a sharp rise in mortality in patients undergoing elective coronary artery bypass grafting. Scand J Clin Lab Invest. (2016) 76:74–81. 10.3109/00365513.2015.1099724 26647957 PMC4720044

[61] Christensson A Grubb A Molvin J Holm H Gransbo K Tasevska-Dinevska G. The shrunken pore syndrome is associated with declined right ventricular systolic function in a heart failure population - the HARVEST study. Scand J Clin Lab Invest. (2016) 76:568–74. 10.1080/00365513.2016.1223338 27622713

[62] González-Rinne A Luis-Lima S Escamilla B Negrín-Mena N Ramírez A Morales A. Impact of errors of creatinine and cystatin C equations in the selection of living kidney donors. Clin Kidney J. (2019) 12:748–55. 10.1093/ckj/sfz012 31584569 PMC6768301

[63] Lentine KL Kasiske BL Levey AS Adams PL Alberú J Bakr MA. KDIGO clinical practice guideline on the evaluation and care of living kidney donors. Transplantation. (2017) 101:S1–S109. 10.1097/TP.0000000000001769 28742762 PMC5540357

[64] Grams ME Garg AX Lentine KL. Kidney-failure risk projection for the living kidney-donor candidate. N Engl J Med. (2016) 374:2094–5. 10.1056/NEJMc1603007 27223153

[65] British Transplantation Society. BTS/RA Living Donor Kidney Transplantation Guidelines (2018). Available online at https://bts.org.uk/wp-content/uploads/2018/07/FINAL_LDKT-guidelines_June-2018.pdf. (accessed February 9, 2019).

[66] Peters AM Perry L Hooker CA Howard B Neilly MD Seshadri N. Extracellular fluid volume and glomerular filtration rate in 1878 healthy potential renal transplant donors: effects of age, gender, obesity and scaling. Nephrol Dial Transplant. (2012) 27:1429–37. 10.1093/ndt/gfr479 22076428

[67] Richardson R Connelly M Dipchand C Garg AX Ghanekar A Houde I. Protocols working group of the canadian blood services' living donation advisory committee: kidney paired donation protocol for participating donors 2014. Transplantation. (2015) 99:S1–S88. 10.1097/TP.0000000000000918 26425842

[68] Organ Procurement and Transplantation Network. Available online at: https://optn.transplant.hrsa.gov/media/1200/optn_policies.pdf#nameddest=Policy_14.

[69] Gaston R Wadström J. Living Donor Kidney Transplantation: Current Practices, Emerging Trends and Evolving Challenges. (1st ed.). CRC Press. (2005). 10.1201/b14909

[70] Organ Procurement and Transplantation Network. Available online at: https://optn.transplant.hrsa.gov/resources/guidance/guidance-for-the-development-of-program-specific-living-kidney-donor-medical-evaluation-protocols/.

[71] Garg N Snyder G Li J Mandelbrot D Poggio ED. Performance of creatinine clearance and estimated GFR in assessing kidney function in living donor candidates. Transplantation. (2020) 104:575–82. 10.1097/TP.0000000000002797 31205262

[72] Gaillard F Courbebaisse M Kamar N Rostaing L Jacquemont L Hourmant M. Impact of estimation versus direct measurement of predonation glomerular filtration rate on the eligibility of potential living kidney donors. Kidney Int. (2019) 95:896–904. 10.1016/j.kint.2018.11.029 30819555

[73] Gaillard F Courbebaisse M Kamar N Rostaing L Del Bello A Girerd S. The age-calibrated measured glomerular filtration rate improves living kidney donation selection process. Kidney Int. (2018) 94:616–24. 10.1016/j.kint.2018.05.016 30143068

[74] Mandelbrot DA Pavlakis M Danovitch GM Johnson SR Karp SJ Khwaja K. The medical evaluation of living kidney donors: a survey of US transplant centers. Am J Transplant. (2007) 7:2333–43. 10.1111/j.1600-6143.2007.01932.x 17845567

[75] Gaillard F Legendre C White CA. GFR Assessment of living kidney donors candidates. Transplantation. (2019) 103:1086–93. 10.1097/TP.0000000000002620 30801521

[76] Garg N Poggio E Mandelbrot D. The evaluation of kidney function in living kidney donor candidates. Kidney360. (2021) 9:1523–1530. 10.34067/KID.0003052021 35373109 PMC8786144

[77] Gaillard F Flamant M Lemoine S Baron S Timsit MO Eladari D. Estimated or measured GFR in living kidney donors work-up? Am J Transplant. (2016) 16:3024–32. 10.1111/ajt.13908 27273845

[78] Huang N Foster MC Lentine KL Garg AX Poggio ED Kasiske BL. Estimated GFR for living kidney donor evaluation. Am J Transplant. (2016) 16:171–80. 10.1111/ajt.13540 26594819

[79] Augustine JJ Arrigain S Mandelbrot DA Schold JD Poggio ED. factors associated with residual kidney function and proteinuria after living kidney donation in the United States. Transplantation. (2021) 105:372–81. 10.1097/TP.0000000000003210 32150042

[80] Ibrahim HN Foley RN Reule SA Spong R Kukla A Issa N. Renal function profile in white kidney donors: the first 4 decades. J Am Soc Nephrol. (2016) 27:2885–93. 10.1681/ASN.2015091018 26888476 PMC5004661

[81] Bergman S Key BO Kirk KA Warnock DG Rostant SG. Kidney disease in the first-degree relatives of African-Americans with hypertensive end-stage renal disease. Am J Kidney Dis. (1996) 27:341–6. 10.1016/S0272-6386(96)90356-X 8604702

[82] Gaillard F Jacquemont L Lazareth H Albano L Barrou B Bouvier N. Living kidney donor evaluation for all candidates with normal estimated GFR for age. Transpl Int. (2021) 34:1123–33. 10.1111/tri.13870 33774875

